# Determinants of self-reported sustained breast cancer screening among women in Shenzhen, China: a study based on the health belief model

**DOI:** 10.3389/fpubh.2026.1936136

**Published:** 2026-09-30

**Authors:** Shuyan Jin, Xiaofeng Ma, Xueyu Yang, Wenxia Zhang, Huan Li, Yuqin Gu, Bo Wu

**Affiliations:** Shenzhen Maternity and Child Healthcare Hospital, Women and Children’s Medical Center, Southern Medical University, Shenzhen, Guangdong, China

**Keywords:** breast cancer screening, China, health belief model, Shenzhen, structural equation model

## Abstract

**Background:**

Breast cancer is the most commonly diagnosed cancer among women worldwide. In Shenzhen, breast cancer screening is available through both a government-funded free program and a range of out-of-pocket or employer-sponsored options; however, evidence on factors associated with sustained screening remains limited. This study aimed to examine the direct and indirect associations of sociodemographic and health-related characteristics and health belief model (HBM) constructs with self-reported sustained breast cancer screening among women in Shenzhen, China.

**Methods:**

A cross-sectional survey was conducted in Shenzhen, China, from November to December 2024, using multi-stage cluster sampling. Data on sociodemographic characteristics, health-related factors, and HBM constructs (five-point Likert scale) were collected. A structural equation model (SEM) was constructed to examine direct and indirect pathways linking sociodemographic characteristics, health-related factors, and HBM constructs to self-reported sustained breast cancer screening. Model fit was assessed using chi-square statistic divided by degrees of freedom (χ^2^/*df*), comparative fit index (CFI), Tucker–Lewis index (TLI), root mean square error of approximation (RMSEA), and standardized root mean square residual (SRMR).

**Results:**

A total of 1,665 women were analyzed; 70.2% (1,169/1,665) had self-reported sustained breast cancer screening. The SEM showed good fit ( χ2/df =3.635; CFI = 0.960; TLI = 0.924; RMSEA = 0.040, SRMR = 0.022). Among the six HBM constructs, cues to action (*β*_standardized_ = 0.106, *p* = 0.013) were positively associated with self-reported sustained breast cancer screening, whereas perceived barriers (*β*_standardized_ = −0.165, *p* < 0.001) were negatively associated with this outcome. Additionally, age (*β*_standardized_ = −0.010, *p* = 0.029), educational level (*β*_standardized_ = 0.020, *p* < 0.001), average monthly income per household (*β*_standardized_ = 0.016, *p* = 0.002), and history of breast diseases (*β*_standardized_ = 0.011, *p* = 0.011) were indirectly associated with self-reported sustained breast cancer screening through perceived barriers. Moreover, history of breast diseases (*β*_standardized_ = 0.013, *p* = 0.028) was additionally indirectly associated with self-reported sustained breast cancer screening through cues to action. Furthermore, age (*β*_standardized_ = −0.003, *p* = 0.045), marital status (*β*_standardized_ = 0.003, *p* = 0.019) and history of breast diseases (*β*_standardized_ = 0.005, *p* < 0.001) were indirectly associated with self-reported sustained breast cancer screening through the sequential pathway involving cues to action and perceived barriers.

**Conclusion:**

Cues to action and perceived barriers were key factors associated with self-reported sustained breast cancer screening. Targeted interventions that strengthen cues to action and reduce perceived barriers may help promote sustained breast cancer screening.

## 1 Introduction

Breast cancer is the most commonly diagnosed malignancy among women worldwide, with approximately 2.3 million new cases and approximately 666,000 deaths in 2022 (1). In China, approximately 357,200 new cases of female breast cancer were diagnosed in 2022, accounting for 15.6% of all female malignancies (2). According to data released by the Shenzhen Center for Chronic Disease Control in 2023, there were 4,252 new cases of female breast cancer, accounting for 16.7% of all female malignancies (3). Shenzhen, a megacity in southern Guangdong Province, China, characterized by a large migrant population from diverse regions across the country, has approximately 17 million permanent residents, including about 7.89 million females. The city features a highly mobile population, with migrants comprising over 65% of residents, and a high level of economic development (4). With ongoing demographic aging, the incidence of breast cancer and its associated disease burden in Shenzhen are expected to increase significantly (5).

Population-based early screening has been consistently shown to improve prognosis and reduce breast cancer-specific mortality (6). Importantly, breast cancer screening is not a one-time event but a continuous process that requires sustained participation to achieve optimal benefits. Previous cohort studies have demonstrated a dose–response relationship between the number of attended screening rounds and breast cancer mortality, with women who participate consecutively across screening rounds experiencing the greatest mortality reduction (7, 8). Furthermore, cohort evidence from Ontario, Canada, has shown that women who fully adhered to recommended biennial mammography screening had a substantially lower risk of breast cancer mortality compared with non-screened women, while partial adherence was also associated with reduced mortality risk (9). These findings suggest that the benefits of breast cancer screening are enhanced by sustained participation over time, rather than isolated screening episodes.

In Shenzhen, breast cancer screening is available through various channels. Since 2009, the government has provided free breast cancer screening every 2 years for female permanent residents aged 35–65 years (10). In parallel, women may undergo screening through employer-sponsored health examinations, self-paid services, or medical and commercial insurance, either as part of proactive health check-ups or opportunistic screening. Together, these multiple screening pathways have contributed to the expansion of breast cancer screening in Shenzhen. The government-funded screening program alone reached an annual screening volume of 267,900 women in 2023, with an early diagnosis rate of 88%, exceeding national urban benchmarks (11, 12). However, despite the expansion of screening services and increased screening uptake, little is known about the factors associated with sustained participation in breast cancer screening among women in Shenzhen. Identifying these factors is therefore important for developing strategies to promote sustained screening and strengthen breast cancer prevention and control efforts.

Although studies from various regions and populations have examined factors associated with breast cancer screening and identified multiple associated factors, including personal health status, socioeconomic characteristics, healthcare accessibility, and psychological factors (13–15), most of these studies have examined ever-screening or screening within a recent interval as the outcome, whereas evidence on factors associated with sustained breast cancer screening remains comparatively limited.

The Health Belief Model (HBM) is one of the most classic theoretical frameworks for explaining and predicting preventive health behavior (16, 17). The model posits that whether an individual adopts a health behavior depends primarily on four types of perception: perceived susceptibility (the perceived likelihood of contracting a disease), perceived severity (the perceived seriousness of the disease), perceived benefits (the perceived advantages of taking action), and perceived barriers (the perceived obstacles to taking action). Self-efficacy, reflecting an individual’s confidence in his or her ability to perform the behavior, was subsequently integrated into the model as an independent construct, and together with the four perceptions it constitutes the core belief constructs of the HBM (18). Within this framework, sociodemographic characteristics and health-related factors are defined as modifying factors that do not directly determine behavior but operate indirectly by influencing individuals’ health beliefs; cues to action (e.g., health education, recommendations from healthcare professionals, reminders from family and friends, and workplace promotion) are considered to trigger health behavior directly (16, 17).

In addition, previous studies have applied the health beliefs to examine repeated mammography participation and adherence to recommended screening schedules (19, 20). These studies demonstrate that health beliefs are significantly associated with sustained screening (19, 20). Therefore, our study used self-reported sustained breast cancer screening as the outcome and extended the classic HBM by incorporating three additional pathways (Figure 1). First, a systematic review of studies conducted among women in mainland China identified sociodemographic characteristics, including age, education, household income, and residential area, as potential factors associated with breast cancer screening participation (21). Given that sociodemographic characteristics and health-related factors may affect individuals’ opportunities and capacity to access screening beyond their health beliefs, we incorporated a direct pathway from sociodemographic and health-related characteristics to sustained breast cancer screening. Second, exposure to cues to action may differ across individuals with different sociodemographic and health-related characteristics. Opportunities to receive health education, recommendations from healthcare professionals, and support from family and social networks may vary across groups with different demographic and health profiles (13). We therefore incorporated a pathway from sociodemographic and health-related characteristics to cues to action. Third, cues to action may not only encourage breast cancer screening participation directly but may also influence screening behavior indirectly by shaping individuals’ health beliefs (22, 23). Accordingly, we incorporated pathways from cues to action to the five HBM belief constructs: perceived susceptibility, perceived severity, perceived benefits, perceived barriers, and self-efficacy. This study aimed to examine the direct and indirect associations of sociodemographic and health-related characteristics and HBM constructs with self-reported sustained breast cancer screening among women in Shenzhen, China, using an extended HBM-based structural equation model (SEM).

**Figure 1:**
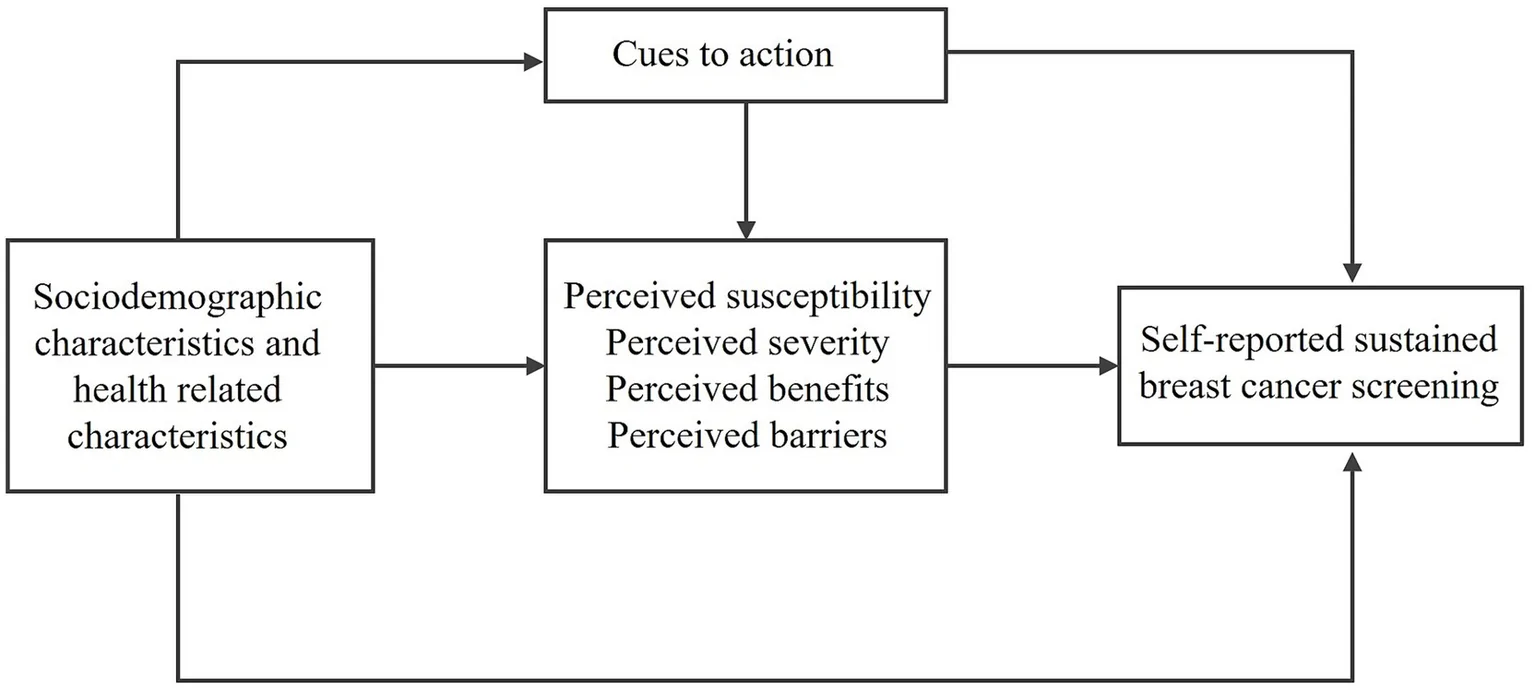
Hypothetical model of this study.

## 2 Materials and methods

### 2.1 Study design and sampling

This cross-sectional survey was conducted in Shenzhen, China, between November 22 and December 2, 2024. The sample size was initially estimated using the standard formula for simple random sampling:

$$n_{0}=\frac{Z_{1-\alpha /2}^{2}P(1-P)}{e^{2}}$$

where $Z_{1-\alpha /2}$ is the standard normal deviate corresponding to a 95% confidence level ( α = 0.05, Z = 1.96), *P* is the expected proportion of women aged 35–64 years who undergo breast cancer screening, set at 0.3778 based on a prior study in Guangdong Province (24), and *e* is the desired margin of error, set at 0.04. Substitution of these values yielded an initial sample size of 565 participants. Because this study employed a multi-stage cluster sampling design, the initial sample size was subsequently inflated by a design effect of 2.0, resulting in a cluster-adjusted sample size of 1,130 participants. Finally, to account for anticipated nonresponse and invalid or incomplete questionnaires, an assumed valid response rate of 80% was applied, yielding a final required sample size of 1,413 participants.

Shenzhen comprises 10 district-level areas across five administrative levels: city → district → subdistrict (street) → community → residential compound. This study employed multi-stage sampling. At the district level, stratified random sampling was used, while convenience sampling was used at lower administrative levels. The 10 administrative districts were stratified into three resource levels based on 2023–2024 per capita GDP: high-resource (i.e., Nanshan, Yantian, and Futian), medium-resource (i.e., Luohu, Dapeng, and Pingshan), and low-resource (i.e., Guangming, Longgang, Longhua, and Bao’an). Five districts were randomly selected, with the number selected from each stratum proportional to stratum size: one district from the high-resource stratum, two from the medium-resource stratum, and two from the low-resource stratum.

Sample sizes were then allocated proportionally to each selected district according to the population distribution of women aged 35–64 years from the 7th National Census, with further proportional allocation by 5-year age groups to reflect the demographic structure. Within each selected district, one subdistrict was conveniently chosen based on fieldwork feasibility and accessibility, followed by selection of 1–5 communities depending on the district-level sample size allocated in the previous stage (<100: 1 community; 100–300: ≤3 communities; >300: ≤5 communities). Finally, one residential compound was conveniently selected per community, where all eligible women residents were invited to participate.

### 2.2 Inclusion and exclusion of participants

Eligible participants were women who (1) were aged 35–64 years; (2) currently resided in the selected residential compounds and (3) were willing to participate and complete the questionnaire. To minimize selection bias, women were excluded if they (1) were receiving medical care at a hospital or community health service center at the time of recruitment, or (2) were visiting a community health service center specifically to receive free breast cancer screening during the recruitment period.

### 2.3 Data collection and quality control

Data were collected electronically using Wenjuanxing^1^, a widely used online survey platform in China. On-site investigators presented a quick response (QR) code to eligible participants in the selected residential compounds, through which participants accessed and completed the questionnaire on their mobile devices.

All investigators had medical backgrounds and received standardized training prior to the survey. Investigators were responsible for recruiting eligible participants and explaining questionnaire items without guiding or suggesting specific answers. Upon completion, questionnaires were submitted directly through the platform, which incorporated built-in data quality checks including completeness validation and duplicate response prevention.

### 2.4 Measures

The questionnaire consisted of three sections: sociodemographic characteristics, health-related factors, and a HBM scale assessing health beliefs. Sociodemographic characteristics included age, body mass index (BMI), place of residence, educational level, marital status, occupation, average monthly income per household, and medical expense payment method.

Health-related characteristics include menopausal status, childbirth history, use of estrogen/hormone replacement therapy, use of estrogen/progesterone-based supplements, use of oral contraceptives, family history of breast cancer (first-degree relatives), history of breast disease, smoking status, drinking status, and dietary preferences for high-fat foods.

Self-reported sustained breast cancer screening was assessed with a single question: participants were asked whether they undergo sustained breast cancer screening (including free and self-paid screening), responding “No” or “Yes” (once a year; once every 2 years; or once every 3 years).

The HBM constructs in this study were measured using 27 items sourced from Champion’s Health Belief Model Scale (CHBMS) and relevant literature on breast cancer screening (25–28). To ensure contextual appropriateness and linguistic clarity for our study population, the wording of items was refined through expert review and focus group discussions. The six measured constructs included (Supplementary Table 1): perceived susceptibility, perceived severity, perceived benefits, perceived barriers, self-efficacy, and cues to action. All items were rated on a five-point Likert scale (1 = strongly disagree, 5 = strongly agree).

### 2.5 Reliability and validity assessment

The internal consistency of each construct was assessed using Cronbach’s alpha coefficients, with values greater than 0.70 considered acceptable. Construct validity was evaluated through confirmatory factor analysis (CFA). Composite reliability (CR) and average variance extracted (AVE) were calculated to assess convergent validity, with CR values above 0.70 and AVE values above 0.50 considered indicative of satisfactory convergent validity. Discriminant validity was assessed using the heterotrait–monotrait ratio of correlations (HTMT) (29).

### 2.6 Statistical analysis

Categorical variables were described using frequencies and percentages, while continuous variables were presented as means and standard deviations. Group comparisons were conducted using the chi-square test for categorical variables and the independent samples *t*-test (or Mann–Whitney *U* test for non-normally distributed data) for continuous variables.

SEM with path analysis was performed to examine the hypothesized relationships in the theoretical framework. Model fit was evaluated using multiple indices: chi-square statistic divided by degrees of freedom ( χ2/df ), comparative fit index (CFI), Tucker-Lewis index (TLI), root mean square error of approximation (RMSEA), and standardized root mean square residual (SRMR). A two-sided *p* < 0.05 was considered statistically significant. All statistical analyses were performed using R software (version 4.4.1).

## 3 Results

### 3.1 Characteristics of participants and univariate analysis

A total of 1,665 participants were included in this study, of whom 70.2% (1169) self-reported sustained breast cancer screening. Among all participants, 826 (49.6%) were aged 35–44 years, and 839 (50.4%) were aged 45–64 years. Regarding educational attainment, 28.3% had bachelor’s degree or above. The majority of participants (94.8%) were married. Detailed information was presented in Table 1.

**Table 1:** Sociodemographic characteristics, health-related characteristics, and HBM constructs among the participants.

Characteristics	Overall (*N* = 1,665)	Do you have sustained breast cancer screenings?		Screening rate (95% CI)	*P*
		No (*N* = 496)	Yes (*N* = 1,169)		
Sociodemographic characteristics					
Age					
35–44	826 (49.6%)	216 (43.5%)	610 (52.2%)	73.8% (70.7, 76.8%)	0.002
45–64	839 (50.4%)	280 (56.5%)	559 (47.8%)	66.6% (63.3, 69.8%)	
BMI	22.82 (3.22)	22.88 (3.50)	22.80 (3.09)	–	0.913
Place of residence					
Low-resource areas	1,090 (65.5%)	363 (73.2%)	727 (62.2%)	66.7% (63.8, 69.5%)	<0.001
Medium-resource areas	309 (18.6%)	76 (15.3%)	233 (19.9%)	75.4% (70.1, 80.0%)	
High-resource areas	266 (16.0%)	57 (11.5%)	209 (17.9%)	78.6% (73.1, 83.2%)	
Educational level					
Junior high school or below	402 (24.1%)	199 (40.1%)	203 (17.4%)	50.5% (45.5, 55.5%)	<0.001
Senior high school or technical secondary school	424 (25.5%)	141 (28.4%)	283 (24.2%)	66.7% (62.0, 71.2%)	
Junior college	367 (22.0%)	84 (16.9%)	283 (24.2%)	77.1% (72.4, 81.2%)	
Bachelor’s degree or above	472 (28.3%)	72 (14.5%)	400 (34.2%)	84.7% (81.1, 87.8%)	
Marital status					
Unmarried	87 (5.2%)	43 (8.7%)	44 (3.8%)	50.6% (39.7, 61.4%)	<0.001
Married	1,578 (94.8%)	453 (91.3%)	1,125 (96.2%)	71.3% (69.0, 73.5%)	
Occupation					
Unemployed or homemaker	126 (7.6%)	52 (10.5%)	74 (6.3%)	58.7% (49.6, 67.3%)	<0.001
Self-employed or farmer	456 (27.4%)	139 (28.0%)	317 (27.1%)	69.5% (65.0, 73.7%)	
Government or public institution employee	342 (20.5%)	63 (12.7%)	279 (23.9%)	81.6% (77.0, 85.5%)	
Enterprise or company employee	379 (22.8%)	95 (19.2%)	284 (24.3%)	74.9% (70.2, 79.2%)	
Retired	362 (21.7%)	147 (29.6%)	215 (18.4%)	59.4% (54.1, 64.5%)	
Average monthly income per household (CNY)					
<3,000	280 (16.8%)	126 (25.4%)	154 (13.2%)	55.0% (49.0, 60.9%)	<0.001
3,001–5,000	518 (31.1%)	153 (30.8%)	365 (31.2%)	70.5% (66.3, 74.3%)	
5,001–8,000	475 (28.5%)	142 (28.6%)	333 (28.5%)	70.1% (65.7, 74.1%)	
>8,000	392 (23.5%)	75 (15.1%)	317 (27.1%)	80.9% (76.5, 84.6%)	
Medical expense payment methods					
No insurance	78 (4.7%)	51 (10.3%)	27 (2.3%)	34.6% (24.4, 46.3%)	<0.001
Urban resident medical insurance	687 (41.3%)	177 (35.7%)	510 (43.6%)	74.2% (70.8, 77.4%)	
Employee medical insurance	764 (45.9%)	188 (37.9%)	576 (49.3%)	75.4% (72.1, 78.4%)	
New rural cooperative medical scheme	125 (7.5%)	74 (14.9%)	51 (4.4%)	40.8% (32.2, 50.0%)	
Commercial insurance	11 (0.7%)	6 (1.2%)	5 (0.4%)	45.5% (18.1, 75.4%)	
Health related characteristics					
Menopausal status					
No	1,166 (70.0%)	290 (58.5%)	876 (74.9%)	75.1% (72.5, 77.6%)	<0.001
Yes	499 (30.0%)	206 (41.5%)	293 (25.1%)	58.7% (54.2, 63.1%)	
Childbirth history					
No	1,453 (87.3%)	421 (84.9%)	1,032 (88.3%)	71.0% (68.6, 73.3%)	0.068
Yes	212 (12.7%)	75 (15.1%)	137 (11.7%)	64.6% (57.7, 71.0%)	
Use of estrogen/hormone replacement therapy					
No	1,561 (93.8%)	481 (97.0%)	1,080 (92.4%)	69.2% (66.8, 71.5%)	0.001
Yes	104 (6.2%)	15 (3.0%)	89 (7.6%)	85.6% (77.0, 91.4%)	
Use of estrogen/progesterone-based supplements					
No	1,594 (95.7%)	482 (97.2%)	1,112 (95.1%)	69.8% (67.4, 72.0%)	0.078
Yes	71 (4.3%)	14 (2.8%)	57 (4.9%)	80.3% (68.8, 88.4%)	
Use of oral contraceptives					
No	1,332 (80.0%)	405 (81.7%)	927 (79.3%)	69.6% (67.0, 72.0%)	0.302
Yes	333 (20.0%)	91 (18.3%)	242 (20.7%)	72.7% (67.5, 77.3%)	
Family history of breast cancer (first-degree relatives)					
No	1,611 (96.8%)	482 (97.2%)	1,129 (96.6%)	70.1% (67.8, 72.3%)	0.631
Yes	54 (3.2%)	14 (2.8%)	40 (3.4%)	74.1% (60.1, 84.6%)	
History of breast diseases					
No	1,051 (63.1%)	377 (76.0%)	674 (57.7%)	64.1% (61.1, 67.0%)	<0.001
Yes	614 (36.9%)	119 (24.0%)	495 (42.3%)	80.6% (77.2, 83.6%)	
Smoking status					
Never smoker	1,628 (97.8%)	477 (96.2%)	1,151 (98.5%)	70.7% (68.4, 72.9%)	0.012
Former smoker	27 (1.6%)	13 (2.6%)	14 (1.2%)	51.9% (32.4, 70.8%)	
Current smoker	10 (0.6%)	6 (1.2%)	4 (0.3%)	40.0% (13.7, 72.6%)	
Drinking status					
Never drinker	1,374 (82.5%)	407 (82.1%)	967 (82.7%)	70.4% (67.9, 72.8%)	0.935
Former drinker	146 (8.8%)	44 (8.9%)	102 (8.7%)	69.9% (61.6, 77.0%)	
Current drinker	145 (8.7%)	45 (9.1%)	100 (8.6%)	69.0% (60.7, 76.2%)	
Dietary preferences for high-fat foods. (e.g., animal fats, fried foods, pastries, sweets, and nuts)?					
No	1,198 (72.0%)	351 (70.8%)	847 (72.5%)	70.7% (68.0, 73.2%)	0.521
Yes	467 (28.0%)	145 (29.2%)	322 (27.5%)	69.0% (64.5, 73.1%)	
HBM constructs					
Perceived susceptibility	9.63 (3.04)	9.01 (2.96)	9.89 (3.03)	–	<0.001
Perceived severity	11.01 (2.49)	11.22 (2.47)	10.92 (2.50)	–	0.018
Perceived benefits	16.90 (2.88)	16.52 (2.96)	17.06 (2.84)	–	<0.001
Perceived barriers	18.07 (6.03)	20.42 (5.68)	17.07 (5.90)	–	<0.001
Self-efficacy	19.87 (3.71)	19.13 (3.83)	20.19 (3.61)	–	<0.001
Cues to action	21.22 (3.68)	20.42 (3.74)	21.56 (3.60)	–	<0.001

HBM, health belief model; BMI, body mass index.

The mean ± standard deviation (SD) scores of perceived susceptibility, perceived severity, perceived benefits, perceived barriers, self-efficacy, and cues to action were 9.63 ± 3.04, 11.01 ± 2.49, 16.90 ± 2.88, 18.07 ± 6.03, 19.87 ± 3.71, and 21.22 ± 3.68, respectively. As shown in Table 1, univariate analysis indicated that participants with self-reported sustained breast cancer screening reported higher mean scores on perceived susceptibility (9.89 vs. 9.01), perceived benefits (17.06 vs. 16.52), self-efficacy (20.19 vs. 19.13), and cues to action (21.56 vs. 20.42) compared to those who did not. In contrast, the self-reported sustained breast cancer screening group had lower mean scores on perceived severity (10.92 vs. 11.22) and perceived barriers (17.07 vs. 20.42). All differences were statistically significant.

### 3.2 Measurement model evaluation

CFA indicated an acceptable fit for the measurement model (χ^2^/*df* = 10.745, CFI = 0.993, TLI = 0.992, RMSEA = 0.077, SRMR = 0.055). All factor loadings exceeded 0.60. Cronbach’s α coefficients ranged from 0.798 to 0.927, and CR values ranged from 0.817 to 0.926, indicating good internal consistency. The AVE ranged from 0.610 to 0.846, supporting adequate convergent validity across all constructs (Supplementary Table 2). Furthermore, all HTMT values remained below the recommended threshold of 0.90, supporting discriminant validity (Supplementary Table 3). Overall, the measurement model exhibited acceptable reliability and validity.

### 3.3 Structural equation modeling

The SEM included five exogenous variables (age, educational level, marital status, average monthly income per household, and history of breast diseases), six HBM constructs as mediators, and self-reported sustained breast cancer screening as the outcome variable. The model demonstrated good fit effects: χ2/df =3.635; CFI = 0.960; TLI = 0.924; RMSEA = 0.040, SRMR = 0.022.

Table 2 presented the direct effects of factors on self-reported sustained breast cancer screening. Cues to action (*β*_standardized_ = 0.106, *p* = 0.013) were positively associated with self-reported sustained breast cancer screening, whereas perceived barriers (*β*_standardized_ = −0.165, *p* < 0.001) showed a negative association. Additionally, being aged 45–64 years (*β*_standardized_ = 0.080, *p* = 0.006), higher educational level (*β*_standardized_ = 0.228, *p* < 0.001), being married (*β*_standardized_ = 0.144, *p* < 0.001), and having a history of breast diseases (*β*_standardized_ = 0.126, *p* < 0.001) were significantly and positively associated with self-reported sustained breast cancer screening.

**Table 2:** Direct effects of structural equation modeling of factors associated with self-reported sustained breast cancer screening.

Dependent variables	Predictor variables	*β*_unstandardized_	*β*_standardized_	*P*
Self-reported sustained breast cancer screening	Perceived susceptibility	−0.003	−0.019	0.469
	Perceived severity	−0.009	−0.047	0.073
	Perceived benefits	−0.007	−0.042	0.277
	Perceived barriers	−0.013	−0.165	<0.001
	Self-efficacy	0.001	0.009	0.800
	Cues to action	0.013	0.106	0.013
	Age	0.073	0.080	0.006
	Educational level	0.091	0.228	<0.001
	Marital status	0.295	0.144	<0.001
	Average monthly income per household	0.012	0.028	0.275
	History of breast diseases	0.119	0.126	<0.001
Cues to action	Age	−0.451	−0.061	0.026
	Educational level	0.031	0.010	0.752
	Marital status	1.242	0.075	0.007
	Average monthly income per household	0.104	0.029	0.295
	History of breast diseases	0.954	0.125	<0.001
Perceived susceptibility	Cues to action	0.155	0.187	<0.001
	Age	−0.466	−0.077	0.004
	Educational level	0.239	0.090	0.003
	Marital status	−0.043	−0.003	0.891
	Average monthly income per household	−0.055	−0.018	0.491
	History of breast diseases	0.238	0.038	0.126
Perceived severity	Cues to action	0.198	0.291	<0.001
	Age	−0.049	−0.010	0.704
	Educational level	−0.153	−0.070	0.012
	Marital status	−0.301	−0.027	0.227
	Average monthly income per household	0.079	0.032	0.214
	History of breast diseases	0.379	0.073	0.002
Perceived benefits	Cues to action	0.586	0.747	<0.001
	Age	0.188	0.033	0.068
	Educational level	−0.031	−0.012	0.522
	Marital status	−0.257	−0.020	0.171
	Average monthly income per household	0.136	0.048	0.006
	History of breast diseases	0.310	0.052	0.002
Perceived barriers	Cues to action	−0.406	−0.250	<0.001
	Age	0.729	0.061	0.017
	Educational level	−0.628	−0.120	<0.001
	Marital status	0.271	0.010	0.629
	Average monthly income per household	−0.559	−0.096	<0.001
	History of breast diseases	−0.840	−0.068	0.004
Self-efficacy	Cues to action	0.695	0.689	<0.001
	Age	−0.046	−0.006	0.746
	Educational level	0.091	0.028	0.181
	Marital status	−0.138	−0.008	0.636
	Average monthly income per household	0.289	0.080	<0.001
	History of breast diseases	0.232	0.030	0.074

Regarding cues to action, being married (*β*_standardized_ = 0.075, *p* = 0.007) and having a history of breast diseases (*β*_standardized_ = 0.125, *p* < 0.001) were positively associated with cues to action, whereas being aged 45–64 years (*β*_standardized_ = −0.061, *p* = 0.026) was negatively associated. For perceived barriers, cues to action (*β*_standardized_ = −0.250, *p* < 0.001), educational level (*β*_standardized_ = −0.120, *p* < 0.001), average monthly income per household (*β*_standardized_ = −0.096, *p* < 0.001), and history of breast diseases (*β*_standardized_ = −0.068, *p* = 0.004) were negatively associated, whereas being aged 45–64 years (*β*_standardized_ = 0.061, *p* = 0.017) was positively associated (Table 2).

The indirect effects of the SEM were shown in Figure 2 and Table 3. Age (*β*_standardized_ = −0.010, *p* = 0.029), educational level (*β*_standardized_ = 0.020, *p* < 0.001), average monthly income per household (*β*_standardized_ = 0.016, *p* = 0.002), and history of breast diseases (*β*_standardized_ = 0.011, *p* = 0.011) were indirectly associated with self-reported sustained breast cancer screening through perceived barriers. Moreover, history of breast diseases (*β*_standardized_ = 0.013, *p* = 0.028) was additionally indirectly associated with self-reported sustained breast cancer screening through cues to action. Furthermore, age (*β*_standardized_ = −0.003, *p* = 0.045), marital status (*β*_standardized_ = 0.003, *p* = 0.019) and history of breast diseases (*β*_standardized_ = 0.005, *p* < 0.001) were indirectly associated with self-reported sustained breast cancer screening through the sequential pathway involving cues to action and perceived barriers.

**Figure 2:**
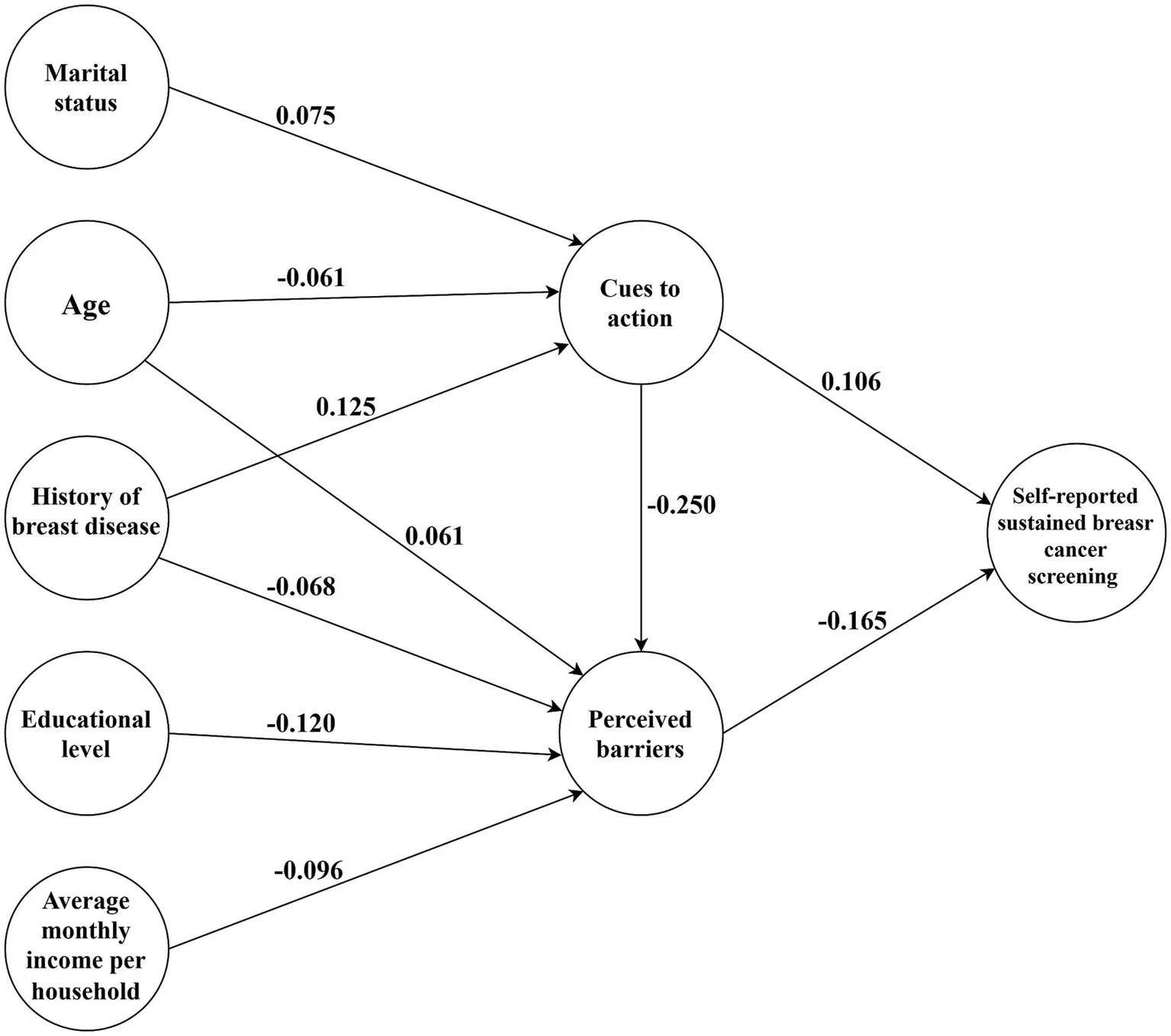
Pathways from sociodemographic and health-related characteristics to self-reported sustained breast cancer screening via health belief model constructs. Values on lines represent standardized path coefficients.

**Table 3:** Indirect effects of structural equation modeling of factors associated with self-reported sustained breast cancer screening.

Mediating pathway	*β*_unstandardized_	*β*_standardized_	*P*
Age → perceived barriers → self-reported sustained breast cancer screening	−0.009	−0.010	0.029
Educational level → perceived barriers → self-reported sustained breast cancer screening	0.008	0.020	<0.001
Average monthly income per household → perceived barriers → self-reported sustained breast cancer screening	0.007	0.016	0.002
History of breast diseases → perceived barriers → self-reported sustained breast cancer screening	0.011	0.011	0.011
History of breast diseases → cues to action → self-reported sustained breast cancer screening	0.013	0.013	0.028
Age → cues to action → perceived barriers → self-reported sustained breast cancer screening	−0.002	−0.003	0.045
Marital status → cues to action → perceived barriers → self-reported sustained breast cancer screening	0.006	0.003	0.019
History of breast diseases → cues to action → perceived barriers → self-reported sustained breast cancer screening	0.005	0.005	<0.001

## 4 Discussion

This study employed an HBM-based SEM to investigate factors associated with self-reported sustained breast cancer screening among women in Shenzhen. The model demonstrated acceptable fit and identified cues to action and perceived barriers as the HBM constructs directly associated with self-reported sustained breast cancer screening. Our findings suggest that targeted interventions focusing on strengthening cues to action and addressing perceived barriers may help promote sustained breast cancer screening.

This study found that perceived barriers were negatively associated with self-reported sustained breast cancer screening. In this study, items measuring perceived barriers encompassed economic burden (Q11, Q16, Q17), psychological concerns regarding adverse outcomes and screening side effects (Q14, Q15), and privacy-related discomfort (Q12, Q13) (Supplementary Table 1). This finding is consistent with evidence from diverse countries and populations, where perceived barriers have been identified as significant impediments to screening behaviors, showing negative associations with both mammography and breast self-examination participation (30–32). Among different perceived barriers types, economic burden has been widely recognized as a major barrier in various studies. Studies from China and the United States have shown that low income, lack of medical insurance, and concerns regarding treatment costs are associated with reduced breast cancer screening participation (33, 34). A systematic review of 67 studies from 22 countries similarly recognized economic burden as the primary barrier (35). Beyond economic factors, the review demonstrated that sociocultural and gender-related factors (i.e., embarrassment/privacy concerns, discomfort with male healthcare providers), and psychological factors (i.e., fear of pain and adverse outcomes, concerns about surgical consequences) also negatively impacted breast cancer screening participation (36), aligning with the multidimensional barriers measured in our study.

Cues to action were positively associated with self-reported sustained breast cancer screening in our study. Items measuring cues to action encompassed public health education, healthcare professional recommendations, family/friends support, and workplace advocacy (Q23–Q27, Supplementary Table 1). Existing evidence supports the effectiveness of these specific cues. A study found that physician/nurse recommendations, family reminders, media campaigns, and community activities effectively promoted screening, with physician/nurse recommendations being the strongest trigger (37). Other studies have similarly demonstrated that physician recommendations, workplace advocacy, and family/social support significantly promote breast cancer screening participation (38, 39).

Previous studies based on the HBM have reported inconsistent associations between HBM constructs and breast cancer screening behavior. Finney Rutten et al. reported that perceived benefits (OR = 1.51) and perceived susceptibility (OR = 1.41) were significantly associated with adherence to annual mammography screening recommendations (20). Hosseini et al. found that self-efficacy is a strong predictor of breast self-examination, clinical breast examination, and mammography among women aged 40 and older in northern Iran (40). Rezaeimanesh et al. (41) found that perceived susceptibility, perceived severity, self-efficacy, and perceived barriers were significantly associated with having ever undergone mammography among women in Tehran. Valenciano-Salas and Juárez-García (42) found that health beliefs differed across mammography adoption stages. Mexican women in the maintenance stage (routinely undergoing mammography at least every 2 years) reported higher levels of perceived benefits and self-efficacy than women in the precontemplation stage (those who had never undergone mammography and had no intention to do so) (42). Our findings indicated that none of the four constructs (i.e., perceived susceptibility, perceived severity, perceived benefits and self-efficacy) was significantly associated with self-reported sustained breast cancer screening. One possible explanation for these differences may be related to Shenzhen’s higher socioeconomic development, well-developed public health resources, and elevated health literacy among residents. Following years of health promotion and education, basic health beliefs—such as perceived benefits and perceived severity—have become widely established at high baseline levels across the population, thereby reducing individual variations and weakening their associations with screening behavior (43). This corresponds with the high self-reported sustained screening rate (70.2%) observed in our survey. In addition, perceived susceptibility may be influenced by prior screening experience: women who have undergone sustained screening over extended periods may develop more stable screening habits, which may attenuate the association between perceived susceptibility and sustained screening behavior (44). Self-efficacy may similarly lack the capacity to differentiate between groups when screening services are readily accessible, as most women perceive themselves as capable of completing a screening—though confidence in a single episode does not necessarily translate into sustained participation at recommended intervals.

Based on these findings, future interventions may benefit from prioritizing the reduction of perceived barriers and the reinforcement of active cues to action, rather than relying solely on general health education. To address perceived barriers, consideration could be given to three potential dimensions: (1) Economic barriers: expanding free screening coverage, offering more flexible scheduling, or exploring mobile and community-based services to reduce logistical and financial hurdles; (2) Psychological barriers: refining health messaging to highlight favorable outcomes associated with early detection and providing accessible support to address screening-related anxiety; and (3) Privacy concerns: enhancing privacy-protected examination settings and accommodating preferences for female healthcare providers where feasible. To strengthen cues to action, multi-level strategies might be explored: (1) Individual level: leveraging personalized digital reminder systems alongside tailored follow-ups by community health personnel; (2) Clinical level: incorporating routine screening recommendations into standard healthcare encounters; and (3) Social level: encouraging workplace-based health incentives and fostering family-supported participation.

This study also examined how sociodemographic and health-related characteristics influence self-reported sustained breast cancer screening indirectly through HBM constructs. A history of breast disease showed the most extensive pathways, operating via cues to action, reduced perceived barriers, and the cues-to-action → perceived barriers sequence, likely because prior healthcare encounters increase receptivity to provider recommendations while reducing psychological resistance to screening (20, 45, 46). Higher educational level and household income provide greater financial and cognitive resources, which help reduce economic and psychological barriers, thereby facilitating self-reported sustained breast cancer screening (33, 39, 47, 48). Age showed a direct positive association with self-reported sustained breast cancer screening but negative indirect effects through perceived barriers and the cues-to-action → perceived barriers sequence. This pattern suggests that while older women may demonstrate higher self-reported sustained breast cancer screening, they might face barriers and receive fewer cues to action, which could partially offset their direct advantage—a complex underlying mechanism warranting further empirical exploration. Marital status was associated with self-reported sustained breast cancer screening through the cues-to-action → perceived barriers pathway, indicating that spousal support may help mitigate perceived obstacles. These findings underscore the need for tailored intervention strategies, suggesting that future public health efforts should focus specifically on these vulnerable or distinct subgroups—particularly older, lower-income, less-educated, unmarried women, and those without a history of breast disease—by aligning interventions with their specific barrier profiles and cue responsiveness.

This study has several strengths. First, the large sample size, combined with balanced coverage across low-resource, medium-resource, and high-resource areas of Shenzhen, enhanced the statistical power and reliability of the findings. Second, unlike most previous studies that focused on whether women had ever participated in breast cancer screening, this study used self-reported sustained breast cancer screening as the outcome variable, which is particularly relevant for economically developed cities like Shenzhen where public health investment is substantial. In such settings, basic screening accessibility has largely been achieved, and the key challenge has shifted from promoting initial screening uptake to fostering sustained participation. Therefore, the findings of this study offer more practical guidance for developing targeted interventions.

However, this study has several limitations. First, the cross-sectional study design limits causal inference regarding the relationships between variables. Second, the outcome variable was based on self-report rather than verified by medical records or objective screening registries, which may introduce recall bias and social desirability bias. Third, although stratified random sampling was used at the district level, convenience sampling was adopted at lower levels, which may have limited the representativeness of the sample. In addition, participants recruited from the same community or residential compound may have shared contextual characteristics, resulting in potential within-cluster correlation, which may have potentially contributed to underestimated standard errors and smaller *p* values. Finally, this study was conducted exclusively in Shenzhen, an economically developed city in China with relatively abundant healthcare resources; therefore, caution should be exercised when generalizing the findings to less developed regions with different healthcare infrastructures and socioeconomic contexts. Future research should employ longitudinal designs to establish causal relationships, use objective screening records to verify self-reported participation, adopt probability-based sampling at all stages of recruitment, and conduct studies in diverse settings to enhance generalizability.

## 5 Conclusion

Cues to action and perceived barriers were key factors associated with self-reported sustained breast cancer screening. Targeted interventions that strengthen cues to action and reduce perceived barriers may help promote sustained breast cancer screening.

## Data Availability

The datasets presented in this article are not readily available because the datasets used in this study are available from the corresponding author upon reasonable request. Requests to access the datasets should be directed to 13823373712@163.com; 13828700253@163.com.

## Footnotes

1
https://www.wjx.cn/
