# Supplementary material for: Determinants of self-reported sustained breast cancer screening among women in Shenzhen, China: a study based on the health belief model
Source: Front Public Health. 2026 Sep 30;14:1936136. doi: 10.3389/fpubh.2026.1936136 (PMC13662284; doi:10.3389/fpubh.2026.1936136)
Supplement: Supplementary file 1 [file Table_1.DOCX]

Supplementary Material

**Supplementary Table 1.** Dimensions and items of the health belief model.

| **HBM constructs** | **Items** |
| --- | --- |
| Perceived susceptibility | Q1. None of my family members have had breast cancer, so I am not at risk of developing it. |
|  | Q2. At my age, I am unlikely to develop breast cancer. |
|  | Q3. I feel physically healthy, so I believe I am not at risk of breast cancer. |
| Perceived severity | Q4. If I develop breast cancer, I might need other treatments in addition to surgery, such as chemotherapy or radiotherapy. |
|  | Q5. If I develop breast cancer, it would have a negative impact on my family. |
|  | Q6. If I develop breast cancer, it might have a negative impact on my job. |
| Perceived benefits | Q7. I believe that breast ultrasound is a good method for early detection of breast cancer. |
|  | Q8. If breast cancer is detected early, the treatment outcomes will be better. |
|  | Q9. I believe that timely diagnosis and treatment of breast cancer can significantly improve a person’s quality of life. |
|  | Q10. I believe that proactively undergoing breast cancer screening can lead to better health outcomes. |
| Perceived barriers | Q11. Screening is meaningless because even if a problem is found, I cannot afford the subsequent medical costs. |
|  | Q12. I feel uncomfortable talking to my doctor about my breast problems. |
|  | Q13. Being examined by medical staff makes me feel uncomfortable. |
|  | Q14. I am afraid to undergo screening because I worry about receiving bad results. |
|  | Q15. I am concerned that screening may expose me to radiation or cause side effects. |
|  | Q16. Breast cancer screening is expensive. |
|  | Q17. If breast cancer screening requires out-of-pocket payment, I would never do it. |
| Self-efficacy | Q18. I can schedule time to get a breast cancer screening. |
|  | Q19. I have appropriate transportation to travel to and from the breast cancer screening facility. |
|  | Q20. I am capable of managing the worries or anxiety related to undergoing breast cancer screening. |
|  | Q21. I can afford the costs associated with breast cancer screening. |
|  | Q22. I have sufficient knowledge about breast cancer screening to make informed decisions. |
| Cues to action | Q23. Public health education on breast cancer screening is available in my area. |
|  | Q24. Healthcare professionals have emphasized to me the importance of breast cancer screening. |
|  | Q25. My family believes that regular screening is an important way to prevent breast diseases. |
|  | Q26. My friends support women undergoing regular breast cancer examinations. |
|  | Q27. My workplace encourages women to schedule breast cancer screenings regularly. |

Abbreviation: HBM, health belief model.

**Supplementary Table 2.** Dimensions and items of the health belief model.

| **Construct** | **Item** | **Loading** | **Cronbach's α** | **CR** | **AVE** |
| --- | --- | --- | --- | --- | --- |
| Perceived susceptibility | Q1 | 0.883 | 0.921 | 0.914 | 0.846 |
|  | Q2 | 0.959 |  |  |  |
|  | Q3 | 0.916 |  |  |  |
| Perceived severity | Q4 | 0.668 | 0.798 | 0.837 | 0.684 |
|  | Q5 | 0.909 |  |  |  |
|  | Q6 | 0.884 |  |  |  |
| Perceived benefits | Q7 | 0.689 | 0.873 | 0.817 | 0.708 |
|  | Q8 | 0.753 |  |  |  |
|  | Q9 | 0.936 |  |  |  |
|  | Q10 | 0.956 |  |  |  |
| Perceived barriers | Q11 | 0.663 | 0.886 | 0.896 | 0.610 |
|  | Q12 | 0.820 |  |  |  |
|  | Q13 | 0.766 |  |  |  |
|  | Q14 | 0.870 |  |  |  |
|  | Q15 | 0.864 |  |  |  |
|  | Q16 | 0.632 |  |  |  |
|  | Q17 | 0.815 |  |  |  |
| Self-efficacy | Q18 | 0.978 | 0.871 | 0.847 | 0.658 |
|  | Q19 | 0.843 |  |  |  |
|  | Q20 | 0.775 |  |  |  |
|  | Q21 | 0.648 |  |  |  |
|  | Q22 | 0.776 |  |  |  |
| Cues to action | Q23 | 0.888 | 0.927 | 0.926 | 0.815 |
|  | Q24 | 0.906 |  |  |  |
|  | Q25 | 0.936 |  |  |  |
|  | Q26 | 0.932 |  |  |  |
|  | Q27 | 0.849 |  |  |  |

**Supplementary Table 3.** Heterotrait–monotrait ratio values for discriminant validity.

|  | **Perceived susceptibility** | **Perceived severity** | **Perceived benefits** | **Perceived barriers** | **Self-efficacy** | **Cues to action** |
| --- | --- | --- | --- | --- | --- | --- |
| Perceived susceptibility | - |  |  |  |  |  |
| Perceived severity | 0.037 | - |  |  |  |  |
| Perceived benefits | 0.205 | 0.603 | - |  |  |  |
| Perceived barriers | 0.600 | 0.083 | 0.317 | - |  |  |
| Self-efficacy | 0.082 | 0.234 | 0.674 | 0.255 | - |  |
| Cues to action | 0.260 | 0.390 | 0.861 | 0.363 | 0.811 | - |
